# Supplementary material for: Inequalities and trends in Neonatal Mortality Rate (NMR) in Ethiopia: Evidence from the Ethiopia Demographic and Health Surveys, 2000–2016
Source: PLoS One. 2020 Jun 10;15(6):e0234483. doi: 10.1371/journal.pone.0234483 (PMC7286487; doi:10.1371/journal.pone.0234483)
Supplement: Attachment — Submitted filename: PONE-D-19-35043_reviewer_dm.pdf [file pone.0234483.s001.pdf]

PLOS ONE

Inequalities and Trends in Neonatal Mortality Rate (NMR) in Ethiopia: Evidence from the Ethiopia Demographic and Health Surveys, 2000-2016

--Manuscript Draft--

**Manuscript Number:**	PONE-D-19-35043
**Article Type:**	Research Article
**Full Title:**	Inequalities and Trends in Neonatal Mortality Rate (NMR) in Ethiopia: Evidence from the Ethiopia Demographic and Health Surveys, 2000-2016
**Short Title:**	Inequalities and Trends in Neonatal Mortality Rate (NMR) in Ethiopia
**Corresponding Author:**	Gebretsadik Shibre<br>Addis Ababa University School of Public Health<br>Addis Ababa, ETHIOPIA
**Keywords:**	NMR; inequality; Ethiopia; DHS
**Abstract:**	Background<br><br>In low- and middle-income countries, substantial inequality in neonatal mortality rates (NMR) remains to the detriment of disadvantaged sub-populations. In Ethiopia, there is a dearth of evidence on the extent and trends of disparity in NMR. This study aims to assess socioeconomic, residence and sex-based inequalities in NMR, and the change over the course of roughly two decades in Ethiopia.Methods<br><br>Using the World Health Organization's (WHO) Health Equity Assessment Toolkit (HEAT) software, data from the Ethiopia Demographic and Health surveys (EDHS) were analyzed between 2000 and 2016. NMR was disaggregated by four equity stratifiers: education, wealth, residence and sex. In addition, absolute and relative inequality measures, namely Difference, Population Attributable Risk (PAR), Ratio, Relative Concentration Index (RCI) and Slope Index of Inequality (SII) were calculated to understand inequalities from wider perspectives. Corresponding 95% Uncertainty Intervals (UI) were computed to measure statistical significance.Findings<br><br>Large educational inequalities in NMR were found in 2000, 2005, and 2011, but wealth-driven inequality remains mainly in 2011. Noticeable sex disparity was noted in all the surveys, and urban-rural differentials in all the surveys except in 2016. While socioeconomic and area-related inequalities had been decreasing over time, sex related inequality did not change during period of study.Conclusions<br><br>NMR appeared to be heavily concentrated among male neonates, among neonates born to illiterate and poor women and those living in rural settings. However, the inequality generally narrowed over time. Interventions appropriate for different sub-populations need to be designed.
**Order of Authors:**	Gebretsadik Shibre
	Dina Idriss-Wheeler
	Sanni Yaya
**Additional Information:**	
**Question**	**Response**
**Financial Disclosure**<br><br>Enter a financial disclosure statement that describes the sources of funding for the work included in this submission. Review the submission guidelines for detailed requirements. View published research	The author(s) received no specific funding for this work

Powered by Editorial Manager® and ProduXion Manager® from Aries Systems Corporation

articles from *PLOS ONE* for specific examples.<br><br>This statement is required for submission and **will appear in the published article** if the submission is accepted. Please make sure it is accurate.<br><br>**Unfunded studies**<br>Enter: *The author(s) received no specific funding for this work.*<br><br>**Funded studies**<br>Enter a statement with the following details:<br>• Initials of the authors who received each award<br>• Grant numbers awarded to each author<br>• The full name of each funder<br>• URL of each funder website<br>• Did the sponsors or funders play any role in the study design, data collection and analysis, decision to publish, or preparation of the manuscript?<br>• **NO** - Include this sentence at the end of your statement: *The funders had no role in study design, data collection and analysis, decision to publish, or preparation of the manuscript.*<br>• **YES** - Specify the role(s) played.<br><br>* typeset	
**Competing Interests**<br><br>Use the instructions below to enter a competing interest statement for this submission. On behalf of all authors, disclose any competing interests that could be perceived to bias this work—acknowledging all financial support and any other relevant financial or non-financial competing interests.<br><br>This statement **will appear in the published article** if the submission is accepted. Please make sure it is accurate. View published research articles from *PLOS ONE* for specific examples.	The authors have declared that no competing interests exist

Powered by Editorial Manager® and ProduXion Manager® from Aries Systems Corporation

**NO authors have competing interests**<br><br>Enter: *The authors have declared that no competing interests exist.*<br><br>**Authors with competing interests**<br><br>Enter competing interest details beginning with this statement:<br><br>*I have read the journal's policy and the authors of this manuscript have the following competing interests: [insert competing interests here]*<br><br>* typeset	
**Ethics Statement**<br><br>Enter an ethics statement for this submission. This statement is required if the study involved:<br><br>• Human participants<br>• Human specimens or tissue<br>• Vertebrate animals or cephalopods<br>• Vertebrate embryos or tissues<br>• Field research<br><br>Write "N/A" if the submission does not require an ethics statement.<br><br>General guidance is provided below. Consult the submission guidelines for detailed instructions. **Make sure that all information entered here is included in the Methods section of the manuscript.**	N/A

**Format for specific study types**<br><br>**Human Subject Research (involving human participants and/or tissue)**<br>• Give the name of the institutional review board or ethics committee that approved the study<br>• Include the approval number and/or a statement indicating approval of this research<br>• Indicate the form of consent obtained (written/oral) or the reason that consent was not obtained (e.g. the data were analyzed anonymously)<br><br>**Animal Research (involving vertebrate animals, embryos or tissues)**<br>• Provide the name of the Institutional Animal Care and Use Committee (IACUC) or other relevant ethics board that reviewed the study protocol, and indicate whether they approved this research or granted a formal waiver of ethical approval<br>• Include an approval number if one was obtained<br>• If the study involved *non-human primates*, add *additional details* about animal welfare and steps taken to ameliorate suffering<br>• If anesthesia, euthanasia, or any kind of animal sacrifice is part of the study, include briefly which substances and/or methods were applied<br><br>**Field Research**<br><br>Include the following details if this study involves the collection of plant, animal, or other materials from a natural setting:<br>• Field permit number<br>• Name of the institution or relevant body that granted permission	
**Data Availability**<br><br>Authors are required to make all data underlying the findings described fully available, without restriction, and from the time of publication. PLOS allows rare exceptions to address legal and ethical concerns. See the PLOS Data Policy and FAQ for detailed information.	Yes - all data are fully available without restriction

A Data Availability Statement describing where the data can be found is required at submission. Your answers to this question constitute the Data Availability Statement and **will be published in the article**, if accepted.<br><br>**Important:** Stating 'data available on request from the author' is not sufficient. If your data are only available upon request, select 'No' for the first question and explain your exceptional situation in the text box.<br><br>Do the authors confirm that all data underlying the findings described in their manuscript are fully available without restriction?	
**Describe where the data may be found in full sentences. If you are copying our sample text, replace any instances of XXX with the appropriate details.**<br><br>• If the data are **held or will be held in a public repository**, include URLs, accession numbers or DOIs. If this information will only be available after acceptance, indicate this by ticking the box below. For example: *All XXX files are available from the XXX database (accession number(s) XXX, XXX.*).<br>• If the data are all contained **within the manuscript and/or Supporting Information files**, enter the following: *All relevant data are within the manuscript and its Supporting Information files.*<br>• If neither of these applies but you are able to provide **details of access elsewhere**, with or without limitations, please do so. For example:<br><br>*Data cannot be shared publicly because of [XXX]. Data are available from the XXX Institutional Data Access / Ethics Committee (contact via XXX) for researchers who meet the criteria for access to confidential data.*<br><br>*The data underlying the results presented in the study are available from (include the name of the third party*	The data underlying the results presented in the study are available from WHO HEAT software webpage: https://www.who.int/gho/health_equity/assessment_toolkit/en/

Powered by Editorial Manager® and ProduXion Manager® from Aries Systems Corporation

and contact information or URL).<br>• This text is appropriate if the data are owned by a third party and authors do not have permission to share the data.<br><br>* typeset	
Additional data availability information:	

Powered by Editorial Manager® and ProduXion Manager® from Aries Systems Corporation

Click here to download Manuscript Inequalities and Trends in Neonatal Mortality Rate.docx

Inequalities and Trends in Neonatal Mortality Rate (NMR) in Ethiopia: Evidence from the Ethiopia Demographic and Health Surveys, 2000-2016

Gebretsadik Shibre[1†], Dina Idriss-Wheeler[2], Sanni Yaya[3,4]

1. Department of Reproductive, Family and Population Health, School of Public Health, Addis Ababa University, Addis Ababa, Ethiopia
2. Interdisciplinary School of Health Sciences, Faculty of Health Sciences, University of Ottawa, Ottawa, Canada
3. School of International Development and Global Studies, Faculty of Social Sciences, University of Ottawa, Ottawa, Canada
4. The George Institute for Global Health, The University of Oxford, Oxford, United Kingdom

[†] **Correspondence to**

GS: gebretsh@gmail.com

Abstract

Background: In low- and middle-income countries, substantial inequality in neonatal mortality rates (NMR) remains to the detriment of disadvantaged sub-populations. In Ethiopia, there is a dearth of evidence on the extent and trends of disparity in NMR. This study aims to assess socioeconomic, residence and sex-based inequalities in NMR, and the change over the course of roughly two decades in Ethiopia.

Methods: Using the World Health Organization's (WHO) Health Equity Assessment Toolkit (HEAT) software, data from the Ethiopia Demographic and Health surveys (EDHS) were analyzed between 2000 and 2016. NMR was disaggregated by four equity stratifiers: education, wealth, residence and sex. In addition, absolute and relative inequality measures, namely Difference, Population Attributable Risk (PAR), Ratio, Relative Concentration Index (RCI) and Slope Index of Inequality (SII) were calculated

to understand inequalities from wider perspectives. Corresponding 95% Uncertainty Intervals (UI) were computed to measure statistical significance.

Findings: Large educational inequalities in NMR were found in 2000, 2005, and 2011, but wealth-driven inequality remains mainly in 2011. Noticeable sex disparity was noted in all the surveys, and urban-rural differentials in all the surveys except in 2016. While socioeconomic and area-related inequalities had been decreasing over time, sex related inequality did not change during period of study.

Conclusions: NMR appeared to be heavily concentrated among male neonates, among neonates born to illiterate and poor women and those living in rural settings. However, the inequality generally narrowed over time. Interventions appropriate for different sub-populations need to be designed.

Key words: NMR; inequality; Ethiopia; DHS

Background

Globally, a staggering 2.5 million newborns died of preventable or treatable causes in 2018 within the first 28 days of life, representing approximately 63% of all infant deaths and 47% of deaths to children five years and under (1). Sub-Saharan Africa (41%) and Southern Asia (37%) account for close to 80% of the 2018 global estimates for neonatal mortality (1) where interventions needed to avoid neonatal

mortality are still low (2-4). Expansions of effective interventions to all who need it improve survivals of neonates, however, launching strategies necessary to meet this aim remain a challenge (5).

Literature shows that socioeconomic inequity in neonatal deaths tend to occur in disadvantaged subpopulations with in a country (5). The majority of neonatal deaths tend to take place in Low and Middle Income Countries (LMIC) (6), disfavoring the poor, illiterate and rural communities (5, 7); neonates born to women in higher socioeconomic positions have better survival rates. However, in most of the LMICs, the inequality in NMR had decreased over the last 20 years (5). As the global community works toward an equity-oriented international agenda to meet the United Nations' Sustainable Development Goals (SDG), it is imperative that interventions reach these subpopulations in order to remove existing NMR disparities. Evidence is necessary to outline the NMR, who is affected most, and how it is changing over time. This, in turn, will inform policies and strategies that target sub-groups of populations who suffer most from high NMR.

In 2018, nearly 100,000 babies died within the first 28 days following birth in Ethiopia (1), contributing to over 50 percent of the under-five mortality rate. McKinnon et al. (2014) flagged Ethiopia as one of the two countries where "wealth related inequality increased by more than 1.5 neonatal deaths per 1000 live births per year between 2000 and 2011" (5, p. 165). The importance of further research in Ethiopia is evident and necessary to understand NMR relative to the various dimensions of inequality (i.e. wealth, education, residence and sex) and how the disparity has changed over the last two decades. In fact, few studies have attempted to assess NMR inequality in Ethiopia

(5, 7, 8). This study enriched the available evidence in a number of areas. First, it investigated NMR inequality through the rigorous method recommended by the World Health Organization (WHO) Handbook on health inequality monitoring. This method considers different dimensions of inequality measures in the analysis to gain a better understanding of the NMR disparity (9). Commonly used absolute (difference) and relative (ratio) simple measures of inequality were calculated to reflect the magnitude of the difference in NMR between studied subgroups and how inequality changed over time (9). In addition to the simple measures, which allow comparison between two sub-groups only, the study also estimated the inequality using measures that account for all sub-groups of an equity stratifier (9). Secondly, it uses the common equity stratifiers recommended by the WHO (9). This is in contrast to most prior studies that limited NMR inequality analysis to just socioeconomic dimensions such as wealth and education (5, 7). Third, most previous studies used traditional regression methods not suitable for inequality studies (8, 10, 11) or were limited to certain specific areas in the country without reflecting aspects of the inequality at the national context (11, 12). Finally, it assessed NMR inequality for the four Ethiopia Demographic and Health Surveys (EDHS 2000, 2005, 2011, 2016) and was not limited to the first and the last EDHS rounds, unlike prior studies (5, 13). This contributed to understanding dynamics of NMR differentials in Ethiopia over roughly two decades. The study particularly sought to answer two questions: 1) what is the status of NMR inequality in Ethiopia across different equity stratifiers?; and 2) how have levels of NMR inequality in different equity stratifiers changed over time between 2000 and 2016?

Methods

Study Setting

With 109 million people, Ethiopia is the most populous country in Africa second only to Nigeria (14). Evidence suggests that Ethiopia experienced consistent economic growth over the last decade, and the agricultural sector has been a major driver of that growth (14). Despite the significant reduction in the proportion of the population living in severe poverty (14), Ethiopia is still one of the poorest nations in the world, with an average per capita Gross Domestic Product (GDP) of approximately US$ 830 in 2017(15). According to the Human Development Index (HDI) ranking, Ethiopia comes in at 174 place out of 188 countries (16), indicating that it is one of the least performing countries with respect to achievements in social and economic dimensions.

Ethiopia suffers the double burden of communicable and non-communicable diseases (NCD). In 2017, communicable, maternal, neonatal and nutritional related diseases were the major causes of deaths, while NCDs were among the foremost problems causing disability (17).

Maternal and neonatal mortalities in Ethiopia are one of the highest globally (1, 18). Although coverage of maternal health services has been on the rise (19), thousands of mothers continue to die from pregnancy related problems, driven partly by the inequitable distribution of services favoring advantaged subpopulations (20, 21). The country prepared and began implementing the 2015-2020 Health Sector Transformation Plan (HSTP) aimed at improving maternal and child health through removal of health disparities (22). Centered on equity of health care, the plan is working towards achieving the Sustainable Development Goals (SDG).

Data sources

Ethiopia Demographic and Health Surveys conducted in 2000, 2005, 2011 and 2016 were used. The methodology DHS follows has been described elsewhere(23-26). Briefly, a two-stage cluster design was employed to select women aged 15 to 49 years. DHS surveys are nationally representative and collect information and data on a wide range of public health related topics and indicators such as maternal health services, child health, maternal and childhood mortality, socioeconomic status, family planning and domestic violence. They were carried out with the financial and technical assistance of Inner City Fund (ICF) International and provisioned through the USAID-funded MEASURE DHS program.

Selection of variables

Inequality is measured for NMR. It is defined as a probability, expressed as deaths per 1000 live births, of a child born in a specific time or period who dies in the first 30 days of life. In the DHS, birth histories collect information on the date of birth and age of death of the neonate. Data on live births that occurred 5 years preceding the surveys were included in the analysis.

Measures

NMR inequality was measured with time over four periods in relation with four equity stratifiers: economic status, education, residence and sex. Economic status was approximated by a wealth index computed based on household assets and characteristics of the household. In DHS, wealth index is computed using Principal

Component Analysis (PCA)(27). It is classified as poorest, poor, middle, rich and richest. Educational status of the mother was categorized as no education, primary and secondary education. Residence is classified as urban vs. rural. Sex is classified as male vs. female.

Statistical analysis

The inequality in NMR was examined in two steps. First, the NMR was disaggregated by the equity stratifiers mentioned above. Secondly, the inequality was assessed using five measures of inequality: Difference, Population Attributable risk (PAR), Ratio, Relative Concentration Index (RCI), and Slope Index of Inequality (SII). While Difference and Ratio are simple measures, the other three are complex measures. Whereas Ratio is a relative measure, the remaining are absolute summary measures. The choice of summary measures is in compliance with evidence suggesting the scientific importance of adopting both absolute and relative summary measures in a single health inequality study (9). The main reason being that, relative and absolute inequality measures could potentially lead to different, even contrasting conclusions (9), and failing to showcase these different scenarios can bias informed decisions. Complex measures account for size of categories of a sub-population, unlike simple measures. When a population shift is likely to occur, especially when trend analysis is an aim in the study, complex measures are likely to reflect the true change in equality over time(9). On the other hand, simple measures are easy for interpretation and understanding. Therefore, an inequality study should combine both simple and complex, as well as relative and absolute measures to provide a more comprehensive analysis.

The WHO's HEAT version 3.1 software was used for the analysis (28). The software is available at:https://www.who.int/gho/health_equity/assessment_toolkit/en/]. Interested researchers can download and use the software. The procedures followed for calculating summary measures are discussed in the HEAT software technical notes (28), and in the WHO handbook on the health inequality monitoring (9). Therefore, only a short account is provided here. For education and economic status, Difference was calculated as NMR in the poorest group minus and NMR in the richest group. For educational status, Difference in NMR between the no education group and secondary education group was conducted. Similarly, for the place of residence, Difference pertains to that between rural and urban populations. PAR was computed as the difference between NMR estimate for the reference subgroup, *yref,* and the national average of NMR. In this study, yref refers to the following to calculate NMR inequality for PAR: rural setting for a place of residence, female for sex, secondary education for education and richest sub-groups for economic status. While zero indicates absence of inequality, the greater absolute value of PAR indicates a higher level of inequality. The SII was computed through a generalized linear regression model. The computation was restricted to two dimensions (education and economic status) and required ranking of a weighted sample of the whole population in order from the most disadvantaged (rank 0) to the most advantaged (rank 1) subgroups. The poorest and uneducated individuals were considered the most disadvantaged, while those that had completed secondary education and the richest subgroups were deemed most advantaged. NMR was regressed against the midpoint value for the wealth or education subgroup and predicted values calculated for those at the two extremes. The difference in the

predicted values between rank 0 and rank 1 produced the SII, representing the difference between lowest and highest while considering all subgroups; positive values mean NMR is more prevalent in the disadvantaged subgroups. Finally, the RCI measures the extent to which NMR is concentrated among the advantaged or disadvantaged groups. If RCI is zero, no inequality in NMR exits. Negative values indicate concentration of NMR among the disadvantaged, while positive values show concentration in advantaged sub-populations.

The change in NMR over time was examined by referring to the 95% Uncertainty Intervals (UI) of the different survey years. When the UIs do not overlap, it implies that there is statistically significant difference in NMR between any two consecutive years. If the UIs overlap, then no inequality exists.

Ethical Consideration

The analyses were completed using the publicly available data from DHS. Ethical procedures were the responsibility of the institutions that commissioned, funded, or managed the surveys. All DHS surveys are approved by ICF international as well as an Institutional Review Board (IRB) in the respective country to ensure that the protocols are in compliance with the U.S. Department of Health and Human Services regulations for the protection of human subjects.

Results

Table 1 presents NMR disaggregated by the four dimensions of inequality for each of the four EDHS. In 2000, the highest NMR was recorded among the poor, middle and rich sub-groups, whereas the lowest was observed among the poorest and richest. This uneven distribution of NMR resulted in an inverted U shape of wealth-related inequality. In 2016, NMR was disproportionately high amongst the rich, with no statistically significant differences between the other four sub-categories. Those who completed secondary education experienced the lowest NMR in 2000 and 2005. However, in 2016, NMR was more evenly distributed across all the three sub-groups of education. Across all surveyed years, a significant gender related disparity persisted with the disadvantage for female neonates. We did not identify a significant difference in NMR between urban and rural areas for all the survey periods. See table 1 for details.

Table [illegible] Neonatal Mortality Rate (deaths per 1000 live births) in Ethiopia disaggregated by Economic Status, Education, Residence and Sex across four time periods (2000, 2005, 2011, 2016).

Inequality dimensions	categories	Survey Years											
		2000			2005			2011			2016		
		Estimate	LB	UB	Estimate	LB	UB	Estimate	LB	UB	Estimate	LB	UB
Economic status	Poorest	41	33	50	39	32	48	50	42	60	36	27	47
	Poor	66	55	79	39	30	49	48	39	59	34	27	42
	Middle	76	63	92	47	39	58	35	28	45	35	27	45
	Rich	61	50	75	45	36	56	39	31	50	47	37	60

	Richest	45	37	54	30	23	40	37	29	48	40	28	57
Education	No-education	62	56	68	41	37	46	46	41	51	39	34	45
	Primary	46	34	62	45	35	57	36	29	43	36	27	46
	Secondary	25	15	41	21	11	38	22	11	42	32	21	50
residence	Rural	60	54	65	41	37	46	43	39	48	38	33	43
	Urban	47	36	60	34	23	52	41	31	53	41	25	67
Sex	Female	49	43	55	33	29	38	34	29	39	26	21	33
	Male	67	60	75	48	42	55	51	45	58	49	42	56

Note: estimate refers to point estimate for NMR; LB=Lower Bound of the estimate; UB=Upper Bound of the estimate

Inequality of NMR was analyzed using simple and complex techniques through the relative and absolute measures as presented in table 2. Wealth (i.e. economic status

equity stratifier) related inequality differed widely by the type of inequality measures. The simple measure of Difference indicated no wealth-driven NMR disparity in all the surveys except in 2011, where, on average, an excess of 13 neonatal deaths per 1000 live births were recorded for the poorest sub-groups. The SII showed that wealth-based inequality remains in 2011 and 2016. The inequality shifted from becoming largely concentrated among the richer sub-groups in 2011 (SII=-18; -28, -9) to be more prevalent among the poorer in 2016 (SII=11; 2.3, 20). The PAR showed marked wealth-related disparity in the favour of wealthier household during the first three surveys where the most disadvantaged sub group experienced higher rates of NMR. In 2000, for instance, on average, the national NMR would have been reduced by 14 neonatal deaths per 1000 live births (PAR= -14; -15, -12) had all the wealth quintiles been on the par with the richest fifth. However, the inequality had significantly declined over time until it disappears in 2016. The RCI measure also confirmed NMR inequality in 2011, with the poor enduring most of the neonatal deaths (Table 2).

Both the relative and absolute measures indicated that NMR appeared to be largely concentrated among infants of women with no formal education. R showed, for instance, that in 2011, NMR among the uneducated women was about 2 times higher than that of women who completed secondary education. PAR and RCI also showed clear disparity in NMR, again disfavoring the illiterate sub-group. By contrast, estimates of the SII revealed that NMR was largely prevalent among women with higher educational attainment in both 2000 and 2011. Overall, educational inequality in NMR had been persistently falling across all measures over time (Table 2).

While no urban-rural differentials of NMR was documented using the simple measures, estimates of the PAR suggest that NMR was more likely to dominate rural settings in the first three time points but disappears in 2016. Other notable finding was the significant sex disparities; NMR was more prevalent among male children in all the surveyed time periods by all the measures (Table 2).

Table 2. Inequality in neonatal mortality rate measured by the different inequality measures across the four dimensions of inequality, between 2000 and 2016 EDHS

		Survey years											
Inequality dimension		2000			2005			2011			2016		
	Measures	Estimate	LB	UB	Estimate	LB	UB	Estimate	LB	UB	Estimate	LB	UB
Economic status	D	-4	-16	8.2	8.9	-3	21	13	0.4	26	-4	-22	13
	PAR	-14	-15	-12	-10	-12	-9	-5	-6.9	-4	0	-1.6	1.6
	R	0.9	0.7	1.2	1.3	0.8	1.8	1.4	0.9	1.8	0.9	0.5	1.3
	RCI	2.1	-1.9	6.1	-1	-6.4	4.2	-7	-12	-2	4.6	-2.6	12
	SII	7.6	-2.9	18	-3	-12	6.5	-18	-28	-9	11	2.3	20
Educatio	D	37	23	50	20	6.2	33	24	8.8	39	6.8	-8.8	22

n	PAR	-33	-36	-31	-20	-22	-17	-21	-24	-18	-6	-8.2	-3
	R	2.5	1.2	3.7	1.9	0.7	3.1	2.1	0.7	3.5	1.2	0.6	1.8
	RCI	-5	-7.7	-2	-1	-4.4	3	-6	-9.6	-2	-2	-7.6	3
	SII	-51	-71	-31	-4	-17	9.1	-27	-39	-14	-9	-20	2.7
Place of residence	D	13	0	26	6.7	-7.9	21	1.9	-9.9	14	-3	-24	18
	PAR	-12	-14	-10	-6	-8.4	-4	-2	-3.2	-0.1	0	-1.9	1.9
	R	1.3	0.9	1.6	1.2	0.7	1.7	1	0.7	1.3	0.9	0.4	1.4
Sex	D	18	9.2	28	15	6.9	22	17	8.7	25	22	14	31
	PAR	-9	-10	-9	-8	-8.2	-7	-9	-9.3	-8	-12	-12	-11
	R	1.4	1.2	1.6	1.4	1.2	1.7	1.5	1.2	1.8	1.8	1.4	2.3

Note: estimate refers to point estimate for NMR; LB=95% lower bound of the estimate; UB=95% upper bound of the estimate.

Discussion

To our knowledge, this is the first attempt to comprehensively evaluate the nature of NMR disparity in Ethiopia over a time period of almost two decades. The findings showed evident inequalities in NMR favoring socioeconomically advantaged

subpopulations (i.e. wealthier and have higher education), female children and those living in urban settings.

Patterns of inequality varied widely by the type of inequality measures analyzed. Estimates of the PAR showed that wealth-related inequalities in NMR had been steadily falling throughout the study period until it disappeared in 2016. Between 2000 and 2011, NMR had reduced, on average, by 9 neonatal deaths per 1000 live births. However, based on the estimates of the SII, the study found huge wealth-related inequality in 2011 and 2016, but not in 2000 and 2005. The inequality significantly elevated in 2011 where the poorest sub-groups were severely disadvantaged when compared with the levels in 2000 and 2005, but it narrowed meaningfully between 2011 and 2016. The RCI similarly demonstrated the wealthy-advantage scenario of NMR in 2011.

Even though findings differed by type of measures, the study confirmed that wealth-driven inequality had generally narrowed overtime. This could largely be due to the gains in the last five or six years in terms of country wide initiatives towards UN SDG 2030. The slowly improved coverage of maternal and child health care services overtime (19), even if the gap in use of these services between the poor and rich persisted or widened (21), suggest a possible contribution to the noticeable drop in the wealth-based inequality. Since 2011, Ethiopia has strengthened its existing strategies for reduction of NMR inequality. In response to the UN SDG call of reducing NMR to 12 deaths per 1000 live birth by 2030 (29), Ethiopia introduced an equity-laden HSTP in 2015 (22). This may have helped the government draw attention to equity of health indicators, including NMR. Concurrently, the region’s economic growth over the last 10

years and the significant fall in the proportion of people living in extreme poverty (14) could be other drivers for decreasing the poor-rich NMR gap overtime.

NMR appeared to be largely concentrated among neonates born to women with no education, coinciding with findings from available literature (5). Educational inequality in NMR either fell steadily or remained unchanged depending on the type of summary measures used. The study identified the year 2016 as the most equitable survey year, where NMR was equally prevalent among the three sub-groups of education by most measures of inequality. A notable exception is that educational inequality in 2000 and 2011 occurred favoring the uneducated subpopulation by the SII estimates. This is at odds with the findings obtained through the other measures and also with prior studies(5). This finding may be anomalous and requires further study.

Large urban-rural disparity in NMR appeared during the first three EDHS time points. However, it had been consistently decreasing throughout the studied years until it disappeared in 2016. Between 2000 and 2011, it was possible to reduce the country's NMR by between 2 to 12 deaths per 1000 live births (on average) had the rural setting been performing on par with the urban areas in terms of neonatal mortality reducing interventions. The disproportionately higher clustering of neonatal mortality reducing interventions (such as access to skilled birth attendants) in urban areas can explain the marked pro-urban profile of NMR in 2000, 2005 and 2011(24-26).The disappearance of inequality by 2016 may be due to improved coverage of these interventions in the rural setting overtime (19).This finding is consistent with prior studies that confirm the pro-urban nature of NMR (30) but not with others (31).

The relative and absolute sex inequalities were notable. NMR was highly prevalent among male children in all the surveyed periods. In 2016, where the sex based disparity was most pronounced, male children experienced an excess of 22 deaths per 1000 live births and endured about twice as much deaths when compared with female children. Unlike the other equity stratifiers based inequality, sex based disparity did not improve with time. Other studies have found similar NMR inequality results with an advantage for female as compared to male neonates (31). Why, then, are male neonates at higher risk of dying than female neonate? Yet another aspect to explore in future studies.

The strength of this study is two-fold. First, inequality in NMR was assessed through different summary measures; adopting a number of inequality measures in the study would help showcase all possible patterns of NMR inequality. Second, the findings use an established high quality WHO monitor database. The database has been prepared by experts proficient in the area and we believe this contributed to the quality of evidence analyzed and reported in this paper.

However, the study also suffered some limitations. It did not identify factors that contribute to the measured NMR inequality. Future studies consider using a decomposition approach to study factors that contribute to the NMR inequality, and to see whether their contribution changes with time. Furthermore, inequality in NMR should be conducted in smaller areas such as villages, towns, districts and zones as the EDHS dataset-based findings cannot be applied to areas smaller than the subnational regions and city administrations.

Conclusions

The study found substantial amount of inequalities in NMR across the different equity stratifiers. Encouragingly, however, NMR inequality in Ethiopia has been on the decline according to almost 20 years of studied data. The improvement in inequality of NMR overtime can be associated with many factors such as Ethiopia's dedication to meet the NMR SDG of 12 deaths per 1000 newborns by 2030, improved economic growth, and improved coverage of maternal and child health services. Inequality studies such as this are useful to inform equity-oriented interventions aimed at eliminating inequality; they signal affected sub groups to strategically target the plan and alleviate the problem. Policy makers should prioritize the subpopulations experiencing the highest NMR without ignoring the whole population. Despite gains made so far, much work is still needed to minimize, if possible eliminate, NMR disparity in across different equity stratifiers.

Acknowledgment

The authors acknowledge WHO for making this equity analysis software freely accessible.

References

1. Levels & Trends in Child Mortality: Report 2019, Estimates developed by the United Nations Inter-agency Group for Child Mortality Estimation, United Nations Children's Fund, New York, 2019.

2. Benova L, Tunçalp Ö, Moran AC, et al Not just a number: examining coverage and content of antenatal care in low-income and middle-income countries BMJ Global Health 2018;3:e000779.

3. Agbessi Amouzou, Meng Ziqi, Liliana Carvajal–Aguirre, and John Quinle. Skilled attendant at birth and newborn survival in Sub–Saharan Africa. Journal of Global Health. 2019

4. WHO. Global Health Observatory Data. Skilled attendants at birth. Available from: https://www.who.int/gho/maternal_health/skilled_care/skilled_birth_attendance_text/en/. Accessed at Nov 22, 2019

5. McKinnon B, Harper S, Kaufman JS, Bergevin Y. Socioeconomic inequality in neonatal mortality in countries of low and middle income: a multicounty analysis. Lancet Glob Health. 2014; 2(3).

6. WHO. Newborns: reducing mortality. https://www.who.int/news-room/fact-sheets/detail/newborns-reducing-mortality, Accessed Oct 23, 2019.

7. Terhi J. Lohela, Robin C. Nesbitt, Juha Pekkanen & Sabine Gabrysch. Comparing socioeconomic inequalities between early neonatal mortality and facility delivery: Cross-sectional data from 72 low- and middle-income countries. Scientific reports, 2019.

8. Mekonnen, Y., Tensou, B., Telake, D.S. et al. Neonatal mortality in Ethiopia: trends and determinants. BMC Public Health 13, 483 (2013) doi:10.1186/1471-2458-13-483.

9. World Health Organization. Handbook on health inequality monitoring with a special focus on low and middle income countries. Geneva: World Health Organization; 2013.

10. Wolde HF, Gonete KA, Akalu TY, Baraki AG & Lakew AM. Factors affecting neonatal mortality in the general population: evidence from the 2016 Ethiopian Demographic and Health Survey (EDHS)—multilevel analysis. BMC Res Notes. 2019 Sep.

11. Mengesha HG, Sahle BW. Cause of neonatal deaths in Northern Ethiopia: a prospective cohort study. BMC Public Health. 2017 Jan.

12. Yaya Y, Eide KT, Norheim OF, Lindtjørn B. Maternal and Neonatal Mortality in South-West Ethiopia: Estimates and Socio-Economic Inequality. PLOS ONE. 2015 Apr; 9(4)

13. Ambel, A.A., Andrews, C., Bakilana, A.M. et al. Examining changes in maternal and child health inequalities in Ethiopia. Int J Equity Health 16, 152 (2017) doi:10.1186/s12939-017-0648-1

14. World Bank. The World Bank in Ethiopia.Available from: https://www.worldbank.org/en/country/ethiopia/overview. Accessed on Nov 11, 2019.

15. International Monetary Fund. World Economic Outlook Database, October 2016. Available from: https://www.imf.org/external/pubs/ft/weo/2016/02/weodata/index.aspx. Accessed Nov 11, 2019.

16. UNDP. Human Development Report 2016. Human Development for Everyone. New York, USA.

17. IHME measuring what matters. Available from :http://www.healthdata.org/ethiopia. Accessed on Nov 11, 2019.

18. Trends in maternal mortality 2000 to 2017: estimates by WHO, UNICEF, UNFPA, World Bank Group and the United Nations Population Division. Geneva: World Health Organization; 2019. Licence: CC BY-NC-SA 3.0 IGO.

19. Ethiopian Public Health Institute (EPHI) [Ethiopia] and ICF. 2019. Ethiopia Mini Demographic and Health Survey 2019: Key Indicators. Rockville, Maryland, USA: EPHI and ICF.

20. Mezmur M, Navaneetham K, Letamo G, Bariagaber H. Socioeconomic inequalities in the uptake of maternal healthcare services in Ethiopia. BMC Health Serv Res. 2017;17:3.

21. Gebre E, Worku A, Bukola F. Inequities in maternal health services utilization in Ethiopia 2000–2016: magnitude, trends, and determinants. Reprod Health. 2018;15(1):119.

22. The Federal Democratic and Republic of Ethiopia Ministry of Health. Health Sector Transformation Plan, 2015-2020. Oct 2015.

23. Central Statistical Agency (CSA) [Ethiopia] and ICF. Ethiopia Demographic and Health survey 2016. Addis Ababa, Ethiopia, and Rockville, Maryland, USA: CSA and ICF; 2016.

24. Central Statistical Agency (CSA) [Ethiopia] and ICF. Ethiopia Demographic and Health survey 2011. Addis Ababa, Ethiopia, and Rockville, Maryland, USA: CSA and ICF; 2011.

25. Central Statistical Agency (CSA) [Ethiopia] and ICF. Ethiopia Demographic and Health survey 2005. Addis Ababa, Ethiopia, and Rockville, Maryland, USA: CSA and ICF; 2005.

26. Central Statistical Agency (CSA) [Ethiopia] and ICF. Ethiopia Demographic and Health survey 2000. Addis Ababa, Ethiopia, and Rockville, Maryland, USA: CSA and ICF; 2000.

27. Rutstein SO and Johnson K. The DHS wealth index. http://www.measuredhs.com/pubs/pdf/CR6/CR6.pdf. Accessed on Nov 23, 2019.

28. Health Equity Assessment Toolkit (HEAT): Software for exploring and comparing health inequalities in countries. Built-in database edition. Version 3.1. Geneva, World Health Organization, 2019.

29. UN General Assembly, Transforming our world : the 2030 Agenda for Sustainable Development, 21 October 2015, A/RES/70/1, available at: https://www.refworld.org/docid/57b6e3e44.html [accessed 25 November 2019].

30. Yi B, Wu L, Liu H, Fang W, Hu Y, Wang Y. Rural-urban differences of neonatal mortality in a poorly developed province of China. BMC Public Health. 2011;11:477.

31. Imtiaz Jehan , Hillary Harris , Sohail Salat , Amna Zeb , Naushaba Mobeen , Omrana Pasha , Elizabeth M McClure , Janet Moore , Linda L Wright & Robert L Goldenber. Neonatal mortality, risk factors and causes: a prospective population-based cohort study in urban Pakistan. Bulletin of the World Health Organization 2009;87:130-138. doi: 10.2471/BLT.08.050963.
