# Supplementary material for: Inequalities and trends in Neonatal Mortality Rate (NMR) in Ethiopia: Evidence from the Ethiopia Demographic and Health Surveys, 2000–2016
Source: PLoS One. 2020 Jun 10;15(6):e0234483. doi: 10.1371/journal.pone.0234483 (PMC7286487; doi:10.1371/journal.pone.0234483)
Supplement: Attachment — Submitted filename: Response to reviewers_f.docx [file pone.0234483.s002.docx]

Dear Editor,

This letter is in reference to your email with reviewers’ comments. We are very pleased that the manuscript is potentially acceptable for publication in PLOS ONE once we have carried out some revisions.

We would like to thank the reviewers for their insightful and helpful comments and for giving us the chance to revise our manuscript. We believe the revised manuscript has been significantly improved and the reviewers’ comments have been addressed adequately. We think in its current form it will make a valuable contribution to the literature on this increasingly important topic.

Please find for your kind consideration the followings: 1) a section-by-section response to the comments and suggestions of the reviewers (below) and 2) the revised manuscript provided as a marked-up copy and a clean copy.

We hope that these changes meet with your favourable consideration. Please do not hesitate to get in touch if you require any further information.

**Response to reviewers**

**Reviewer #1:** The manuscript is well written and well presented. The analysis is perfect. It can be published with or without my suggestions. I have made one simple suggestion to add the overall NMR in one row in table-1.

**Response:** We would like to thank the reviewer for the thoughtful comments and for taking the time to read our manuscript.We agree with the suggestion made which has been applied.

Reviewer #2: The manuscript sheds light on an important topic. The current format of the manuscript was not suitable for review as there is not page or line numbers. My following major comments refer to page/paragraph numbers based on the submission document:
**Response:** Thanks for pointing out this mistake. This has now been revised.

**Introduction:**
1. p.11, lines 13‒15: Authors need to elaborate the statement “(previous) regression methods not suitable for inequality studies”, especially when HEAT itself uses regression models for estimating RII and SII.

**Response:** The reviewer’s point is valid. We have now revised the statement.

Results:
2. p.22, Table 2: The table is hard to read and needs proper notes for abbreviated forms if inequality measures.

**Response:** The suggestion of the reviewer was considered and applied.

3. Authors may consider using charts, which would be useful to compare the confidence intervals for categories over time.

**Response**: We thank the reviewer for this relevant suggestion. While drafting the results, we tried to present main findings through charts and/or line graphs. But a major issue has prevented us from doing so. In fact, in the line graphs, the confidence intervals for the different categories (dimensions of inequality) when plotted on same x-y plane are too close to each other and nearly override one another so that readers cannot draw any useful information (see figure below). If we opt to present confidence intervals for the different categories separately, then we would have several charts/graphs. This is not effective and is not in line with the journal requirements. In the new figure below, we presented here a line graph that shows the wealth and education related disparities using SII measure. In 2005 and 2011, intervals for wealth and education are passing over the other and make it difficult to separate out the individual intervals. The problem would even be more complex if we had added intervals for the other categories (residence, sex). Thus, we strongly believe that a table is a better way to highlight the disparities across the various dimensions.

|  | wealth | edu |
| --- | --- | --- |
| 2000 | 2.1 | -5 |
| 2005 | -1.1 | -0.7 |
| 2011 | -6.9 | -5.9 |
| 2016 | 4.6 | -2.3 |

Fig. wealth and education-based NMR disparity

**Discussion:**
4. p.23‒24: Does your findings support the first para of discussion section “The findings showed evident inequalities in NMR favoring socioeconomically advantaged subpopulations (i.e. wealthier and have higher education), female children and those living in urban settings”? If you compare the confidence intervals, NMR disparity has remained similar over time for all the dimensions except education.

**Response:** The suggestion of the reviewer was considered. In this first paragraph, we only tried to describe whether NMR inequality remains in all dimensions of inequality examined in the study. We did not attempt to discuss whether inequalities changed over time as this is clearly discussed in other paragraphs of the discussion section. The question whether or not confidence intervals overlap is important when exploring changes over time, rather than examing whether disparity existed in the first place. For this latter case, showing that the intervals do not contain the null value is sufficient. For PAR, D, RCI and SII measures, the null value is 0. So, any intervals computed from these measures that crossed 0 shows no inequality. For the R, the null value is 1 and any interval obtained from this measure that crossed 1 do show absence of inequality. This is how we interpreted our findings and is in line with what was described in our methods section.

To show that your concern was addressed in other paragraphs of the discussion, we discussed inequality for each dimension separately below to show whether these disparities increased or decreased over time and whether intervals overlap or not:

**Wealth-based disparity**: wealth related disparity appeared by Difference and RCI in 2011 only and we could not talk problem of overlap. By SII, it appeared in 2011 and 2016. Since inequalities are negative in 2011 (-28, -9) and positive in 2016 (2.3, 20), these two confidence intervals do not overlap at all. There is no way for negative and positive intervals to overlap. The PAR showed inequality in 2000, 2005 and 2011 with confidence intervals respectively (-15, -12), (-12, -9) and (-6.9, -4). As we can see, they do not overlap. The only exception is that -12 is both the end of the first interval and is as the same time the starting point for second interval. But, this does not constitute overlap of the intervals. If for instance the second interval is (-14, -11), then we can subjectively declare that the two intervals overlap as they have substantial number of points in common.

**Residence-based disparity**: we showed residence related inequality based on the PAR measure for the years 2000, 2005 and 2011. The intervals are (-14, -10), (-8.4, -4) and (-3.2, -0.1) respectively. As we can see, these three intervals are not overlapping.

**Sex-based disparity**: we showed sex disparity of NMR by all the three measures. Based on PAR for instance, the four intervals for the year 2000-2016 respectively are -10, -9; -8.2, -7; -9.3,-8 and -12, -11.Intervals in 2000 and 2011, and 2005 and 2011 have only slight overlap. But, intervals in 2000 and 2005& in 2000 and 2016 do not overlap. So as we move from 2000 to 2016, it increased overall.

5. The findings of this study clearly indicates towards the ‘inverse equity hypothesis,’which states that as countries scale up use of health services from initially low levels, early increases in inequality could be expected—if the hypothesis holds true, after the initial widening, inequalities in these countries should begin to narrow. The authors may want to review papers by Victora et al. (2000) [doi: 10.1016/S0140-6736(00)02741-0] and Cutler, Deaton &Lleras-Muney (2006) on this issue.

**Response:** We agree with this suggestion and the manuscript was revised accordingly.

6. The authors appear to miss some important articles while comparing their findings with existing studies. I’d recommend to review papers by Onarheim et al. (2012) [doi:10.1371/journal.pone.0041521]; Skaftun, Ali &Norheim (2014) [doi:10.1371/journal.pone.0106460]; and Ambel et al. (2017) [doi: 10.1186/s12939-017-0648-1]; among others.

**Response:**The suggestion of the reviewer was considered and applied.

7. p.24, para 3: “Even though findings differed by type of measures, the study confirmed that wealth driven inequality had generally narrowed…. suggest a possible contribution to the noticeable drop in the wealth-based inequality.” This statement needs to be supported by evidence. Given the starting period(s), country-wide initiatives towards UN SDG or HSTP are unlikely to affect study’s findings.

**Response:** The suggestion of the reviewer was considered and applied.

8. An aim of the HEAT software is to compare inequity over time/countries and disseminate guidance for the policymakers and public health practitioners to address the disparities. I would strongly recommend to include a discussion on policy and programmatic implications of the study findings. for this reason, the authors may want to refer to the following published papers: Marchant et al. (2019) [doi: 10.1503/cmaj.190219]; Knippenberg et al. (2005) [doi: 10.1016/S0140-6736(05)71145-4]; Steven et al. (2019) [doi: 10.4314/ejhs.v29 i6.3]; Onarheim et al. (2012) [doi:10.1371/journal.pone.0041521].

**Response** :Thanks for this excellent suggestion which has now been applied.
